# Paresthesia as an Initial Presentation of Hansen’s Disease in a Non-endemic Setting

**DOI:** 10.7759/cureus.109634

**Published:** 2026-05-25

**Authors:** Kritika Reddy

**Affiliations:** 1 Family Medicine, University of California Los Angeles, Los Angeles, USA

**Keywords:** cutaneous paresthesia, dermatitis, hansen’s disease, leprosy, manifestations of leprosy

## Abstract

Leprosy or Hansen’s disease is an infectious disease caused by the intracellular organism *Mycobacterium leprae (M. leprae).* The disease has a low prevalence in the United States and follows an indolent course with symptoms often manifesting several years after initial exposure. Reaching a diagnosis of Hansen's disease can often be challenging in a non-endemic setting. Additionally, reaching a diagnosis in the absence of skin symptoms is difficult, as the diagnostic method that provides the most clinical reliability is a skin biopsy or slit skin smear (SSS). Through this skin test, the acid-fast bacillus *M. leprae* is visualized.

We present a case of a 55-year-old woman from California who initially presented with neuropathy and continued to suffer from three years of progressive sensory and motor neuropathies before the diagnosis of Hansen’s disease. She lived in a non-endemic setting but was likely exposed through her work in medical tourism in Playa del Carmen, Mexico. A diagnosis was ultimately reached once she presented with skin lesions that allowed for a biopsy. This case also highlights the challenge in reaching a timely diagnosis of Hansen’s disease, which, due to its low prevalence in North America, can often be overlooked in a differential diagnosis.

## Introduction

Leprosy, also known as Hansen's disease, is caused by the intracellular organism *Mycobacterium leprae (M. leprae)* [1]. It primarily affects the skin and peripheral nerves, with skin manifestations being one of the early signs of the disease [2]. Despite the World Health Organization (WHO) declaration in 2005 that the disease had been eliminated [3], new cases continue to manifest. The disease, being endemic primarily to Brazil, Nepal, India, and Indonesia [3], can decrease clinical suspicion for practitioners in countries outside of endemic regions. It is also important to note that the time from exposure to *M. leprae* to clinical manifestations can range from 5-10 years, as the bacteria have a prolonged incubation period [4]. Making a timely diagnosis can be challenging as the disease typically has an indolent course, and the symptoms of Hansen’s disease frequently mimic other, more common conditions, including other infectious diseases, neurologic, or autoimmune diseases. A timely diagnosis is crucial, as the subsequent delay to treatment can often result in debilitating complications and decreased quality of life.

In this case, we discuss a patient who presented with worsening neurologic symptoms over three years. She was initially recommended to undergo a neurologic evaluation for radiculopathy. Upon pursuing cervical and lumbar magnetic resonance imaging (MRI), electromyography (EMG), and nerve conduction study (NCS), it was determined that her symptoms were less likely related to radiculopathy. As part of the initial neurologic evaluation, a positive Lyme disease IgM titer, which was later determined to be a false-positive, further stymied her diagnostic course. We did not consider Hansen’s disease as a likely diagnosis until she developed skin manifestations, which prompted a skin biopsy; the biopsy revealed *M. leprae*. The delay in confirming the diagnosis of Hansen’s disease, unfortunately, led to having to manage the type 1 immunologic reaction before she was able to start anti-mycobacterial therapy.

## Case presentation

**Figure 1:**
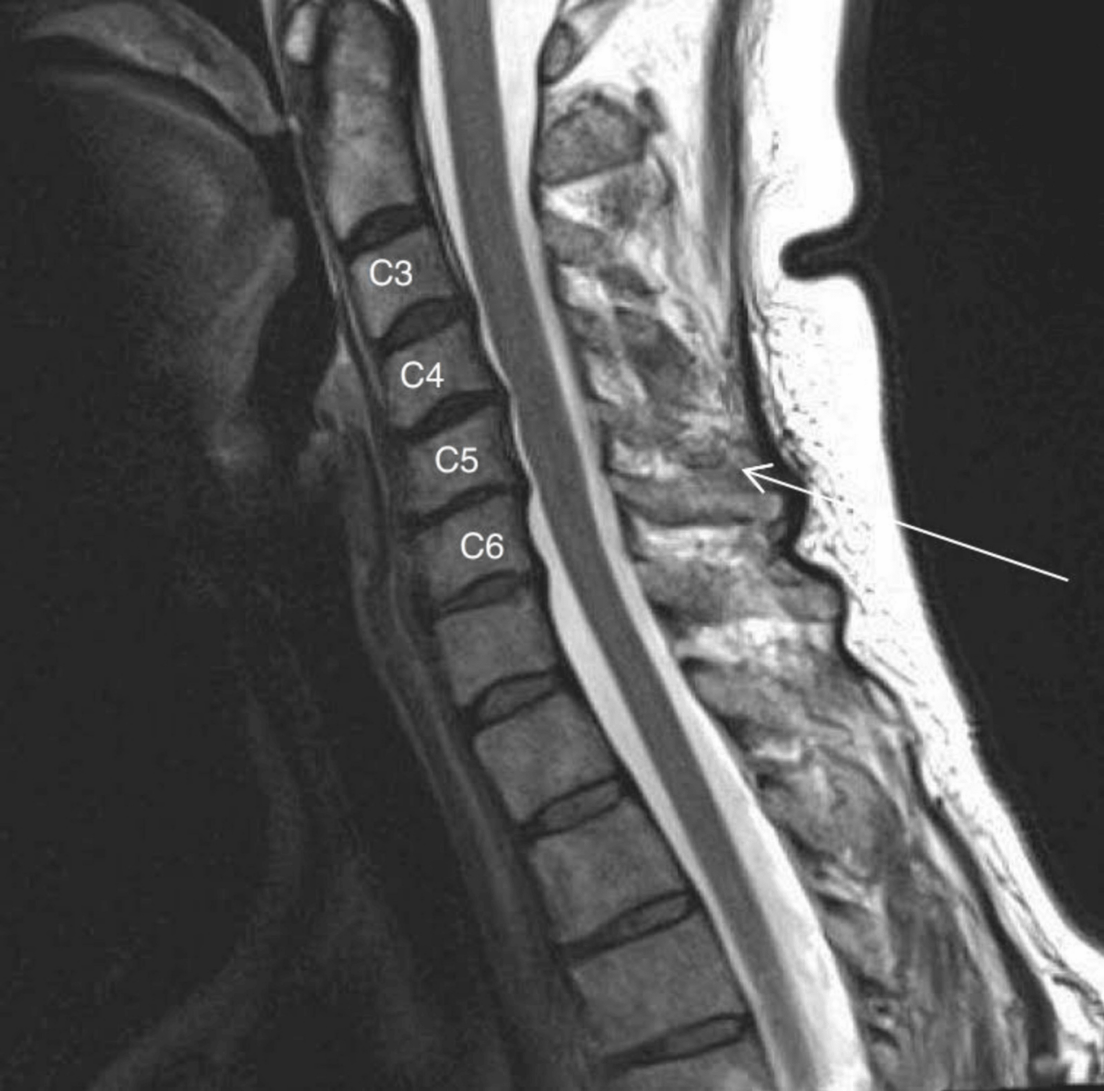
Cervical MRI showing foraminal stenosis (arrow) at the C5-C6 level MRI: magnetic resonance imaging

**Figure 2:**
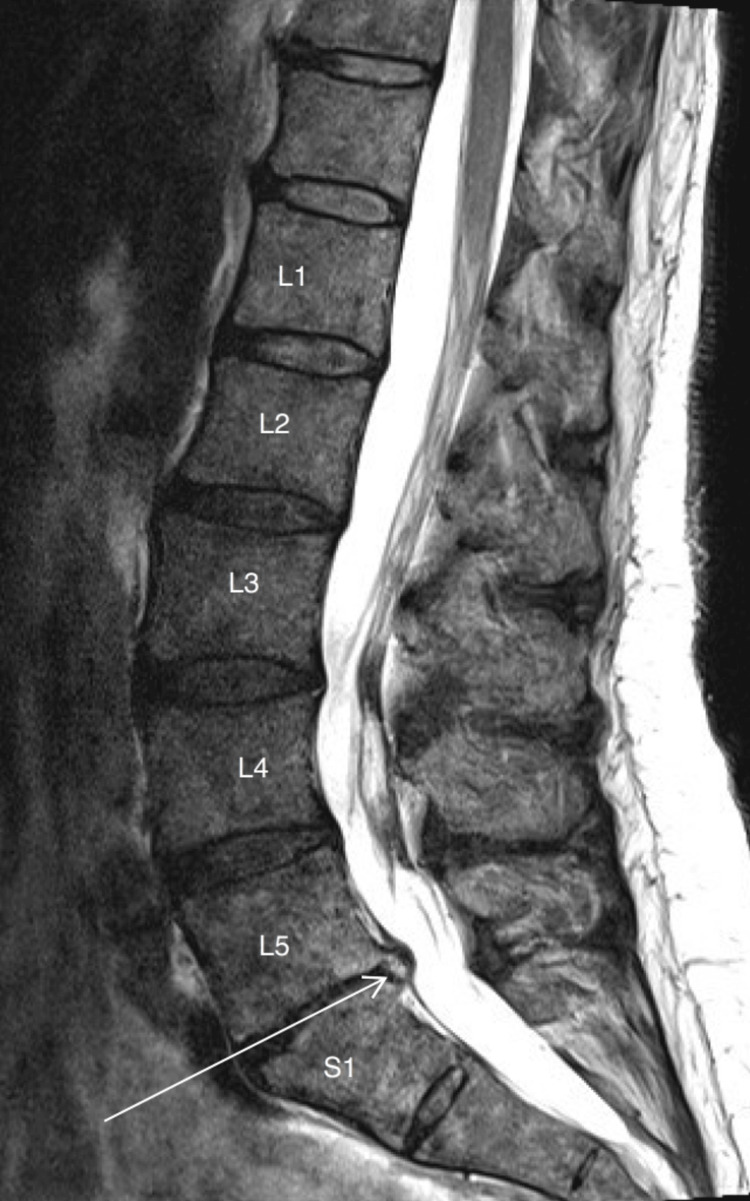
Lumbar MRI showing the osteophyte complex (arrow) at the level L5-S1 MRI: magnetic resonance imaging

A 55-year-old woman with no significant past medical history presented with paresthesias on the right side of her face, nose, lips, ear, upper extremity, and thumb. She also noted lower back soreness with paresthesias in both feet to the mid-shin. These symptoms had been ongoing for the past three years. Her symptoms worsened at night and seemed to improve with stretching and movement. On examination, facial sensation was intact, with diminished light touch from both shins to the feet. She was also noted to have an unsteady gait with a positive Romberg test. She completed a routine lab panel for neuropathy (including a test for Lyme titers), cervical MRI, lumbar MRI, and EMG with NCS. Cervical MRI results showed moderate left foraminal stenosis at C5-C6 (Figure 1), and lumbar MRI results showed multilevel degenerative changes and an osteophyte complex at L5-S1, without significant spinal canal or foraminal stenosis at any lumbar level (Figure 2).

The lab tests revealed a positive IgM Lyme serology, negative IgG Lyme titer, and a vitamin B6 deficiency (Table 1). 

**Table 1:** Pertinent results from neurologic and rheumatologic evaluation ELISA: enzyme-linked immunosorbent assay; ANCA: anti-neutrophil cytoplasmic antibody; Sm/RNP: Smith and ribonucleoprotein; C3: complement C3; C4: complement C4; C1q: complement component 1q; ESR: erythrocyte sedimentation rate; CRP: c-reactive protein; CK: creatine kinase; HBs Ag: hepatitis B surface antigen; HCV Ab: hepatitis C virus antibody; MTB-QuantiFERON: *Mycobacterium tuberculosis* complex; LIV: Lyme Index value

Test	Patient’s result	Reference range
Vitamin B6	17 nmol/L	20-125 nmol/L
*Borrelia burgdorferi* Abs total, by ELISA	1.5 LIV	0.0-1.20 LIV
*B. burgdorferi* Ab, IgM by immunoblot	Positive	Negative
*B. burgdorferi* Ab, IgG by immunoblot	Negative	Negative
Cryocrit	Negative	Negative
C-ANCA	<1:20	<1:20
P-ANCA	<1:20	<1:20
Proteinase-3 Ab	<20.0	<20.0
Myeloperoxidase Ab	<20.0	<20.0
Sm/RNP antibodies	<20/<20 units	<20 units
C3	100 mg/dL	76-165 mg/dL
C4	24 mg/dL	14-46 mg/dL
C1q complement component	110 µg/mL	109-242 µg/mL
ESR	12 mm/h	<25 mm/h
CRP	<0.3 mg/dL	<0.8 mg/dL
Uric acid	5.1 mg/dL	2.9-7.0 mg/dL
Protein/creatinine ratio, urine	0.1	0-0.4
CK, total	38 U/L	38-282 U/L
HBs Ag	Nonreactive	Nonreactive
HCV Ab screen	Nonreactive	Nonreactive
MTB-QuantiFERON ELISA	Negative	Negative

**Figure 3:**
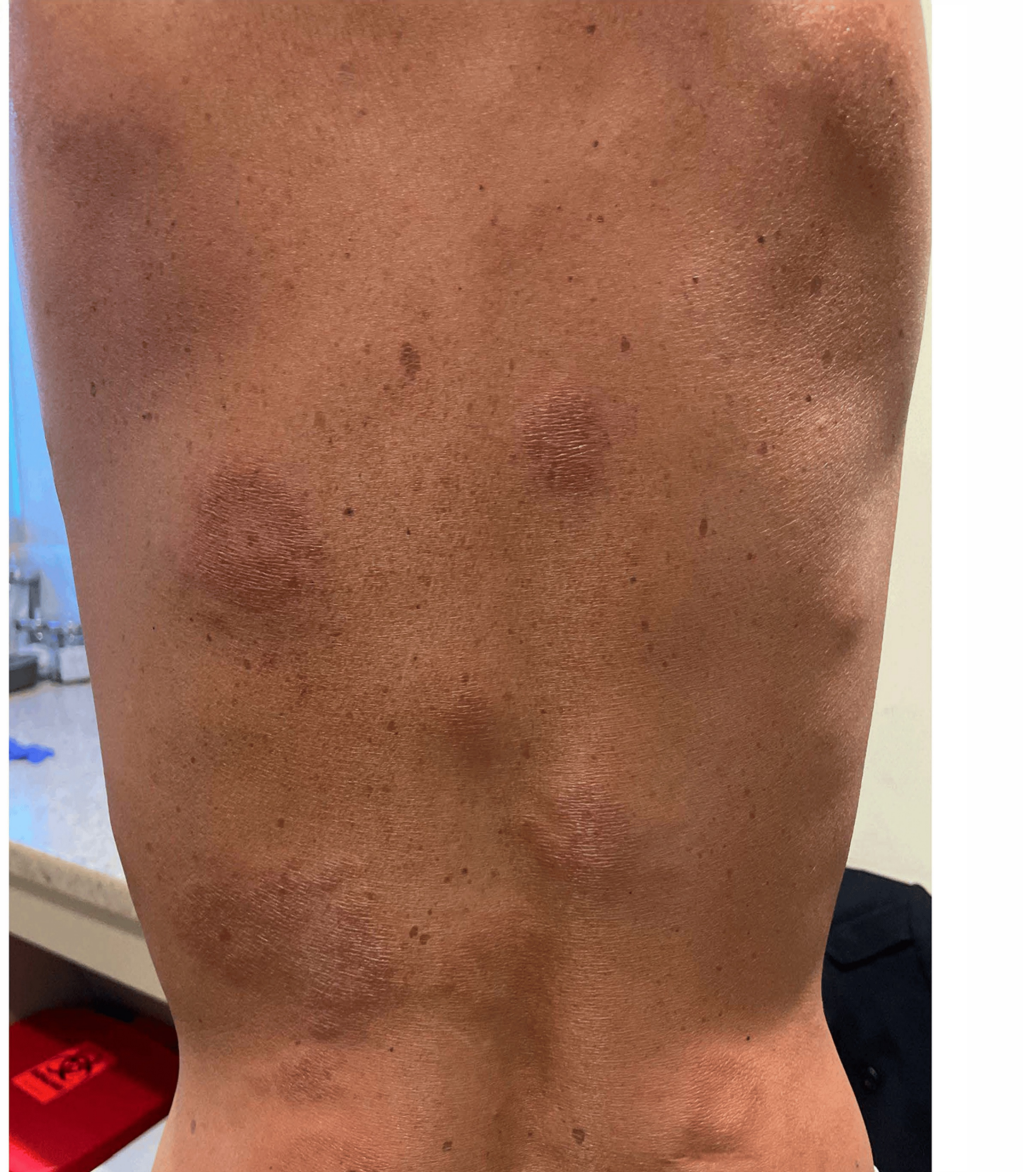
Erythematous, indurated, and hyperpigmented rash on the back

She was then instructed to follow up with an infectious disease specialist to discuss the positive IgM Lyme titer and was started on a Vitamin B6 supplementation. A few days later, she developed a patchy rash which began on the chest and arm and then spread to the face, arms, legs, and back (Figure 3). This rash appeared hyperpigmented and indurated with urticaria.

Upon gathering further history and evaluation with an infectious disease specialist, it was noted that the patient works in medical tourism and is often based in Playa del Carmen, Mexico. It was determined that the IgM Lyme titer was likely a false-positive, as the duration of her symptom course in conjunction with a negative Lyme IgG titer was not consistent with an active Lyme disease infection. She also completed EMG/NCS testing, which was consistent with mononeuritis multiplex; these results were not consistent with a demyelinating neuropathy, and there was no electrodiagnostic evidence of lumbosacral or cervical radiculopathy. After consulting her neurologist regarding the rash, she was recommended to seek evaluation with a rheumatologist. Her neurologist was concerned that the granulomatous appearance of the rash could be concerning for vasculitis. The rheumatologic evaluation included the following serologies: cryocrit, anti-neutrophil cytoplasmic antibody (ANCA), Smith and ribonucleoprotein (Sm/RNP), C3, C4, C1q, C1Q binding assay, erythrocyte sedimentation rate (ESR), c-reactive protein (CRP), uric acid (UA), urine protein creatinine (UPC), creatine kinase (CK), hepatitis B/C (Hep B/C), and QuantiFERON Gold. These results were all negative (Table 1). 

**Figure 4:**
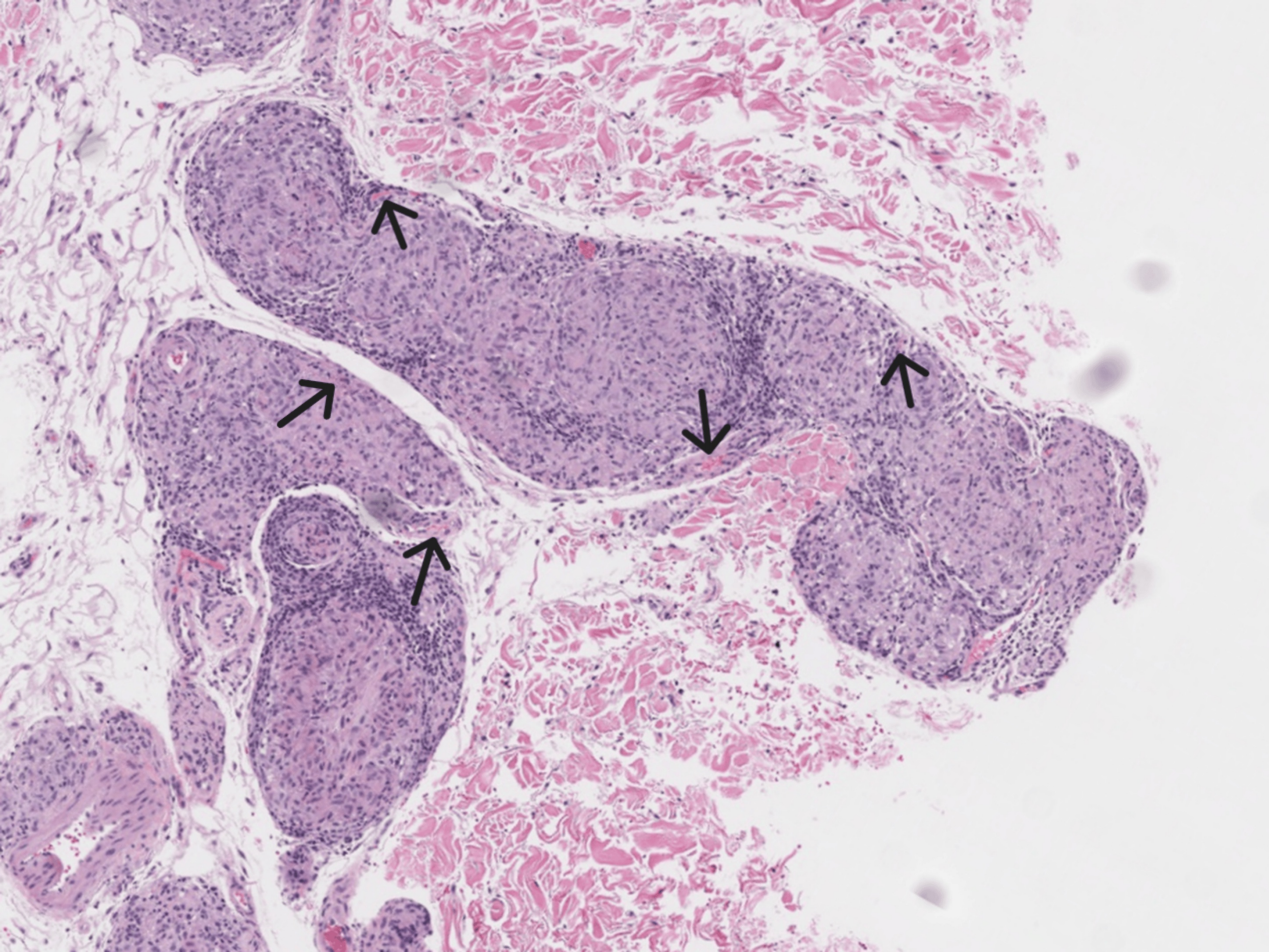
Presence of Mycobacterium leprae (arrows) from skin biopsy using the Ziehl-Neelsen stain (acid-fast stain)

As the rheumatologic evaluation was unrevealing, she was then recommended to follow up with a dermatologist to rule out vasculitis. A punch biopsy was taken from the medial chest and left breast at the site of the rash. The initial biopsy report showed the presence of numerous acid-fast mycobacteriaand was negative for pathogenic fungal organisms (Table 2). The biopsy specimen was then sent for further testing to clarify the species of *Mycobacterium*; the finalized biopsy report confirmed *Mycobacterium leprae *(Figure 4)*.* 

**Table 2:** Skin biopsy results AFB: acid-fast bacilli; GMS: Grocott-Gömöri's methenamine silver stain

Clinical information
A-B. Rule out urticarial vasculitis versus subacute lupus versus other vasculitides
Final diagnosis
A. Skin, chest-medial (punch biopsy):
- Superficial/deep granulomatous dermatitis with scattered plasma cells and rare neutrophils (see comment)
- AFB/Fite special stains highlight numerous acid-fast mycobacteria
- GMS appears negative for pathogenic fungal organisms
- Colloidal iron highlights increased dermal mucin
- No vasculitis or malignancy in the initial and deeper sections examined
B. Skin, left breast (punch biopsy):
- Superficial/deep granulomatous dermatitis with scattered plasma cells and rare neutrophils (see comment)
- AFB/Fite special stains highlight numerous acid-fast mycobacteria
- GMS appears negative for pathogenic fungal organisms
- Colloidal iron highlights increased dermal mucin
- No vasculitis or malignancy in the initial and deeper sections examined

While waiting for the biopsy report to be finalized, the patient developed heightened edema in the face, hands, and feet, as well as worsening paresthesia in the scalp, ears, and upper and lower extremities. Additionally, she experienced constant eye watering and redness, loss of facial muscle strength, and was unable to squeeze her eyes shut. The facial thickening was thought to be consistent with a type 1 immunologic reaction, and she was recommended to start on immunosuppression with 60 mg prednisone once daily. The use of steroids was recommended to quiet the immune response from causing further nerve injury with the goal of initiating anti-mycobacterial therapy once the immune response was under better control. She was later started on an anti-mycobacterial regimen after consultation with the National Hansen’s Disease Program (NHDP). Lastly, she was recommended to follow up with ophthalmology to be evaluated for lagophthalmos.

## Discussion

This case presents the opportunity to discuss a unique presentation of Hansen’s disease in which isolated neurologic symptoms precede a rash. It is difficult to reach a diagnosis of leprosy until a rash appears, as skin biopsy or slit skin smear (SSS) remains the validated diagnostic test [4,5]. Other reliable diagnostic methods to evaluate for *M. leprae* before the appearance of a rash are limited [5]. Though *M. leprae* can also be detected in the nasal mucosa of exposed individuals, its presence alone does not predict transmission or clinical susceptibility [5]. More recently, serologic testing to detect PGL-1, the major surface antigen specific to *M. leprae*, has become available [6]. However, this remains an auxiliary diagnostic test in suspected cases [6].

This patient’s case also demonstrates the challenge in reaching a diagnosis in a non-endemic setting, as leprosy is not usually suspected as a likely differential. The symptom course of the disease is indolent [4], and the initial presentation can often be associated with other etiologies [1]. As was the case with this patient, her symptoms had been ongoing for three years before their worsening prompted evaluation by several specialists. Her symptoms, as determined by evaluation from multiple specialists, seemed to be more likely attributed to several common differentials, including cervical radiculopathy, lumbar radiculopathy, vasculitis, or other infectious disease. Additionally, her paresthesia symptoms responding to Vitamin B6 supplementation in conjunction with a likely false-positive Lyme IgM titer argued against an infectious etiology. Even when the rash presented, she was recommended to seek further evaluation from a rheumatologist. It is important to note that Hansen’s disease can also present as vasculitis, which often makes a diagnosis of rheumatologic disease seem likely [1,7]. Furthermore, immunologic reactions in leprosy can frequently mimic other autoimmune conditions [8]. Only when the rheumatologic serologies were unrevealing was she recommended to seek further evaluation with a dermatologist. The biopsy completed from the dermatology evaluation then confirmed the diagnosis of leprosy. It was not unreasonable for the specialists she saw to follow this diagnostic path, though not considering leprosy as a differential can often be the biggest hurdle to reaching a diagnosis [4]. Additionally, misdiagnosis can be a significant barrier to detection and treatment and can be common even in endemic settings [9].

Throughout the evaluation, leprosy was likely overlooked because it remains rare in the United States. In the early 1990s, only 136-187 cases were reported every year [5]. However, in non-endemic countries, leprosy can still be observed in individuals who have spent a significant amount of time in an endemic region [10]. This patient’s social history of working in medical tourism and spending considerable time in Playa del Carmen (an area known to be populated with armadillos - a common zoonotic host [4,11]) should have been an essential piece in considering leprosy as a diagnosis. The prolonged path to diagnosis, unfortunately, led to a delay in the treatment and progression of her symptoms, including increased debility.

## Conclusions

This case highlights the challenging and often non-linear path to confirming the diagnosis of leprosy in a non-endemic region. As she initially presented with neuropathy as opposed to skin manifestations, her diagnosis was stymied by other, more common differentials. She underwent several months of evaluation and testing from multiple specialists. Although thorough, the circuitous path of this evaluation delayed treatment and led to a complication: a type 1 immunologic response. She eventually received treatment with oral steroids followed by the appropriate antimycobacterial therapy.
